# The thermoregulatory theory of yawning: what we know from over 5 years of research

**DOI:** 10.3389/fnins.2012.00188

**Published:** 2013-01-02

**Authors:** Andrew C. Gallup, Omar T. Eldakar

**Affiliations:** ^1^Department of Ecology and Evolutionary Biology, Princeton UniversityPrinceton, NJ, USA; ^2^Center for Insect Science, University of ArizonaTucson, AZ, USA

**Keywords:** brain cooling, contagious yawning, sleep, thermoregulation, yawning

## Abstract

Over the past 5 years numerous reports have confirmed and replicated the specific brain cooling and thermal window predictions derived from the thermoregulatory theory of yawning, and no study has found evidence contrary to these findings. Here we review the comparative research supporting this model of yawning among homeotherms, while highlighting a recent report showing how the expression of contagious yawning in humans is altered by seasonal climate variation. The fact that yawning is constrained to a thermal window of ambient temperature provides unique and compelling support in favor of this theory. Heretofore, no existing alternative hypothesis of yawning can explain these results, which have important implications for understanding the potential functional role of this behavior, both physiologically and socially, in humans and other animals. In discussion we stress the broader applications of this work in clinical settings, and counter the various criticisms of this theory.

## Introduction

It was recently discovered that contagious **yawning** frequency varies with seasonal climate variation (Gallup and Eldakar, 2011). Pedestrians in Tucson, Arizona, an arid desert climate in the Western United States, completed a survey in which they viewed a series of yawning photos and answered questions about their own yawning behavior while doing so. Two independent samples of participants were collected, one during the early summer when temperatures averaged 37°C (equivalent to human body temperature) and the other during the winter when temperatures averaged 22°C (equivalent to room temperature). Results showed that pedestrians in the winter condition were nearly twice as likely to yawn during the survey and that even when accounting for variables such as the amount of sleep the night before, duration of time spent outside prior to being surveyed, and relative humidity, ambient temperature was the only significant predictor of yawns. Specifically, participants were more likely to yawn contagiously at lower ambient temperatures across both seasonal conditions. Furthermore, the proportion of individuals yawning in the summer quickly dropped as the length of time spent outside increased, suggesting that yawns were suppressed following an initial acclimation to sudden temperature changes.

Although these results may seem unexpected, they match the specific predictions of the thermoregulatory theory that were initially proposed over 5 years ago (Gallup and Gallup, 2007). Since then, a growing body of both descriptive and experimental research has demonstrated a clear and consistent association between yawning and brain **thermoregulation** in homeotherms (Gallup, 2010). Alongside the repeated demonstration of predicted brain temperature fluctuations surrounding yawning in rats (*Rattus norvegicus*) (Shoup-Knox et al., 2010; Shoup-Knox, 2011), a fundamental principle of this theory concerns the specific predictions associated with yawning frequency and ambient temperature variation (i.e., the thermal window hypothesis), which directly follow models of body temperature regulation among **homeotherms** (e.g., Scholander et al., 1950). Research on humans, non-human primates, rats and birds shows that yawning frequency is altered by fluctuations in ambient temperature (Campos and Fedigan, 2009: *Cebus capucinus*; Deputte, 1994: *Macaca fascicularis*; Gallup et al., 2009, 2010b: *Melopsittacus undulatus*; Gallup et al., 2011: *Rattus norvegicus*; Gallup and Eldakar, 2011: *Homo sapiens*), and in all cases the specific correlation between temperature and behavior remains consistent with the thermoregulatory theory. Following an overview of this theory, we briefly outline the clinical and medical literature revealing a strong association between abnormal yawning and thermoregulatory dysfunction in humans (Gallup and Gallup, 2008; Ghanizadeh, 2011). Lastly, we address the attempts to dismiss or undermine the empirical support of this theory (Elo, 2010, 2011; Guggisberg et al., 2010, 2011; Walusinski, in press) and show how they have remained untenable.

## The brain cooling hypothesis

The fact that yawning is elicited by numerous stimuli has contributed to the lack of consensus regarding its function (Miller et al., 2012a), and new hypotheses for yawning continue to emerge (e.g., Thompson Cortisol Hypothesis, Thompson and Bishop, 2012). Perhaps the most common belief is that yawning functions to increase oxygen levels in the blood. However, Provine et al. (1987) disproved this hypothesis by showing that breathing heightened levels of oxygen or carbon dioxide did not influence yawning, although it did increase breathing rates. They also demonstrated that physical exercise sufficient to double breathing rates had no effect on yawning. Therefore, contrary to popular belief, yawning does not serve a respiratory function and is controlled by a separate mechanism from breathing.

A vast amount of literature suggests that yawns function to stimulate or facilitate arousal during state change (Baenninger, 1997; also see state change hypothesis: Provine, 1986, 1996, 2005). Although there is conflicting evidence from studies measuring an arousal response accompanying yawning (e.g., Laing and Ogilvie, 1988; Regehr et al., 1992; Sato-Suzuki et al., 1998; Kita et al., 2000; Kasuya et al., 2005; Guggisberg et al., 2007), across vertebrate taxa yawning occurs in anticipation of important events and during behavioral transitions. Further research shows that yawning precedes modified activity levels in humans and non-human primates (Baenninger et al., 1996; Giganti et al., 2002; Vick and Paukner, 2009: *Pantroglodytes*). Evidence from endocrine, neuotransmitter, and pharmacological mechanisms also supports the view that yawning is an important mediator of behavioral arousal levels (Baenninger, 1997). Accordingly, it has been proposed that yawning functions to modify levels of cortical arousal during variable environments. Branching from this theory, it has recently been postulated that it is the effect of yawning on brain thermoregulation that produces these adaptive benefits of arousal and mental efficiency (Gallup, 2010; Gallup et al., 2011).

The thermoregulatory theory was initially developed on the view that the physiological consequences of a yawn could provide the necessary components needed for effective brain cooling in homeotherms (Gallup and Gallup, 2007). Specifically, it proposed that yawning functions as a compensatory cooling mechanism. These predictions have since been echoed in continued research investigating the physiological consequences of yawning in humans (Shoup-Knox, 2011; Corey et al., 2012). Consistent with this theory, pharmacological research demonstrates that yawning is under the control of the hypothalamus (Argiolas and Melis, 1998), a brain structure strongly linked to thermoregulation (Cooper, 2002).

### Mechanisms of thermoregulation and how yawning cools the brain

Brain temperature of homeotherms is determined by three variables: the temperature of arterial blood flow, the rate of this blood flow, and the metabolic heat production (Baker, 1982). The localized circulatory changes accompanying yawning alter these first two variables. Physiological cooling mechanisms that influence blood supplying the brain include **convective heat transfer**, thermal conduction, and **evaporative heat loss**. To date, three main processes have been described for how yawning could cool brain temperature in mammals and birds. Below we apply them specifically to humans.

First, yawning produces significant changes in circulation, including acceleration in heart rate (Heusner, 1946) and elevation of blood pressure (Askenasy and Askenasy, 1996). More specifically, powerful jaw stretching during yawning produces increases in neck, head, and facial blood flow (Zajonc, 1985; Askenasy, 1989), and the deep inspiration during yawning produces significant downward flow in cerebrospinal fluid and an increase in blow flow in the internal jugular vein (Schroth and Klose, 1992). The network of veins within the lateral pterygoid muscle has been described to operate as a peripheral pump, and the powerful and extended contraction of these muscles during yawning acts to squeeze blood from the associated plexus (Bhangoo, 1974). Brain temperature is consistently 0.2°C higher than that of arterial blood supplying the brain (Kiyatkin et al., 2002), and that on average, 0.66J of heat energy per minute per gram is released from the brain through blood flow (Yablonskiy et al., 2000). As a result, the aforementioned processes during yawning act like a radiator by removing hyperthermic blood from brain and simultaneously introducing cooler blood from the lungs and extremities, thereby cooling cortical surfaces through convection.

Secondly, it is hypothesized that yawning also provides a direct heat exchange from the deep inhalation of cooler ambient air (Gallup et al., 2009). The air exchange during yawning would cool venous blood draining from the nasal and oral orifices into the cavernous sinus, which surrounds the internal carotid artery supplying blood to the rest of the brain (Zenker and Kubik, 1996). Again, this would provide a cooling effect through convection. Furthermore, it has recently been discovered that pharyngeal cooling rapidly and selectively decreases brain temperature in primates, and tympanic temperature in humans, by cooling the carotid arteries (Takeda et al., 2012).

Third, it has recently been discovered that the posterior wall of the maxillary sinus serves as an origin for both medial and lateral pterygoid muscle segments (Koritzer and Hack, 2002), which allows the thin sinus walls to flex when the pterygoid musculature contracts during jaw activity such as yawning. This powerful flexing of the sinus walls has been proposed to ventilate the human sinus system similar to that described in birds (Sedlmayr and Witmer, 2001), providing yet another mechanism for cerebral cooling by yawning in humans. Accordingly, yawning could reduce brain temperature by ventilating the sinus system and promoting the evaporation of the sinus mucosa (Gallup and Hack, 2011).

### The inhibition of yawning following nasal breathing and forehead cooling

The first empirical support for the brain cooling model came over 5 years ago when Gallup and Gallup (2007) discovered that contagious yawning can be modulated by different breathing and forehead temperature manipulations in humans. It has been well documented that behaviors such as nasal breathing and forehead cooling effectively result in brain temperature reductions (Mariak et al., 1999; Harris et al., 2007; Zenker and Kubik, 1996), and when presented with a contagious yawning stimulus, participants performing these behaviors showed a reduced tendency to yawn. Thus, it was proposed that cerebral cooling from these conditions inhibited the mechanisms that normally trigger contagious yawning. Consistent with this interpretation, mechanical introduction of cool air into the nasal cavity can lower brain surface temperature (Harris et al., 2007), and further research using intracranial thermocouples demonstrated that nasal inhalation can produce rapid reductions (0.1°C/min) in frontal lobe brain temperature as well (Mariak et al., 1999). Likewise, forehead cooling reduces the temperature of the blood in cutaneous and subcutaneous venous plexuses, which is then transported through venous communications into the venous plexuses of the dura mater (Zenker and Kubik, 1996). Forehead cooling also enhances dissipation of heat from the skull, and the cooling of venous blood by the skin can in turn cool the carotid arterial blood supply to the brain through convective heat transfer. More recently, these same methods of brain cooling (i.e., nasal breathing and forehead cooling) have been shown to either prevent or delay bouts of excessive yawning in patients suffering from thermoregulatory dysfunction (Gallup and Gallup, 2010).

### Predicted fluctuations in brain and oral temperature surround yawning events in rats and humans

The fundamental predictions of the thermoregulatory theory are that yawning should (1) be triggered by brain **hyperthermia**, and (2) produce a measureable cooling effect (Table 1). To explicitly investigate the relationship between yawning and brain temperature, Shoup-Knox et al. (2010) took continuous measures of prelimbic brain temperature in rats using thermocoupled temperature probes and paired these recordings to confirmed yawning events. Consistent with these predictions, yawning was triggered in response to rapid increases in brain temperature (0.11°C), and followed by equivalent decreases in temperature immediately thereafter. Furthermore, all increases in brain temperature during this study of ≥0.1°C corresponded to yawning and stretching behavior, and all rapid decreases in temperature of ≥0.5°C occurred during the first minute following yawns only.

**Table 1:** **Empirical tests of the brain cooling hypothesis**.

**Prediction**	**Confirmed**	**Species (area)**
(1) Rises in brain temperature should precede the onset of yawns	– Gallup and Gallup (2010)	– Humans (oral temperature)
	– Shoup-Knox et al. (2010)	– Rats (prelimbic cortex)
	– Shoup-Knox (2011)	– Rats (prelimbic cortex)^*^
(2) Decreases in brain temperature should follow yawns	– Gallup and Gallup (2010)	– Humans (oral temperature)
	– Shoup-Knox et al. (2010)	– Rats (prelimbic cortex)
	– Shoup-Knox (2011)	– Rats (prelimbic cortext)^*^ (preoptic area)

* *Indicates a replication of this effect in an independent sample of this species*.

Shoup-Knox (2011) further strengthened this relationship by measuring temperature changes in both the prelimbic cortex and preoptic area of the hypothalamus of rats before and after yawns. Temperature recordings from the prelimbic cortex replicated the relationship from previous findings (Shoup-Knox et al., 2010). However, the temperature relationship in the preoptic area differed slightly. In particular, cooling following yawns was delayed in the preoptic area in comparison to the prelimbic cortex, and there was no increase in temperature in the preoptic area prior to yawns. Therefore, while both internal and external brain tissues cooled after yawns, only the cortex showed an increase in temperature leading up to this response.

Predicted temperature fluctuations have also been confirmed using non-invasive temperature measures in humans. Gallup and Gallup (2010) described two case studies of women suffering from chronic bouts of yawning, in which one could anticipate the onset of these bouts and record her oral temperature before and after each episode. Both patients complained of unpredictable and uncontrolled yawning attacks that lasted up to 45 min in length, occurring between one to fifteen times per day. These episodes involved repeated deep and overwhelming yawns that caused their eyes to water and their nose to run. Each yawn consisted of powerful jaw, neck, and body stretching, and time spent in between yawns included deep inhalations. Both women also reported feeling cold during or after each bout of excessive yawning, and often experienced goose bumps and shivering as a consequence. The woman who took oral temperature recordings reported the onset of each bout occurring during mild hyperthermia (37.5°C), and that there was an average decrease in temperature of 0.4°C immediately following each episode (Gallup and Gallup, 2010).

## Thermal window hypothesis

Homeothermic species preserve a relatively constant body temperature as ambient temperature fluctuates, using a combination of autonomic and behavioral mechanisms controlled by the central nervous system (Bicego et al., 2007). According to the thermoregulatory theory, the mechanisms triggering yawning should also be controlled by ambient temperature variation (Gallup and Gallup, 2007, 2008). The predictions derived from this theory, also known as the thermal window hypothesis, follow other behavioral models of temperature regulation among homeotherms (Scholander et al., 1950). Therefore, yawning should be influenced by both the direction and range of ambient temperature.

The thermal window hypothesis has three primary predictions (Table 2). First, yawns should increase in frequency with initial rises in ambient temperature. As ambient temperatures rise, so do temperatures in the brain (Shoup-Knox, 2011) stimulating thermoregulatory mechanisms to control temperatures within a normal range. Second, yawns should decrease as ambient temperatures draw near or exceed body temperature. Since the temperature of the ambient air gives a yawn its cooling utility, yawning at ambient temperatures above body temperature would be counter productive, and thus more effective evaporative cooling mechanisms (e.g., sweating, panting) should be triggered during extreme heat stress. A third prediction states that yawning should diminish when temperatures fall below a certain point, because countercurrent heat exchange could potentially cool the brain below optimal homeostasis. The specific tests of the first two predictions are outlined below, while the third and final prediction has yet to be formally investigated.

**Table 2:** **Empirical tests and observations supporting the thermal window hypothesis**.

**Prediction**	**Confirmed**	**Species**
(1) Yawning should increase with initial rises in ambient temperature	– Campos and Fedigan (2009)	– Capuchins (*Cebus capucinus*)
	– Deputte (1994)	– Macaques (*Macaca fascicularis*)
	– Gallup et al. (2009, 2010b)	– Budgerigars (*Melopsittacus undulatus*)^*^
	– Gallup et al. (2011)	– Rats (*Rattus norvegicus*)
(2) Yawning should decrease when ambient temperatures draw near or exceed body temperature	– Gallup et al. (2009)	– Budgerigars (*Melopsittacus undulatus*)
	– Gallup et al. (2011)	– Rats (*Rattus norvegicus*)
	– Gallup and Eldakar (2011)	– Humans
(3) Yawning should diminish at very low ambient temperatures (e.g., −10°C)	– Unknown	– None tested

* *Indicates a replication of this effect in an independent sample of this species*.

The thermal window hypothesis was first tested in budgerigars, an arid zone parrot. Gallup et al. (2009) measured yawning and other thermoregulatory behaviors in three distinct temperature conditions: control (22°C), an increasing range (22–34°C), and a high range (34–38°C). Consistent with the theory, yawning was indeed more frequent during the increasing range, but began to decrease in frequency at the high temperature range when evaporative cooling mechanisms became more frequent (i.e., panting). These results directly match Scholander et al.'s (1950) model of temperature regulation among avian species. Their model assumes that birds expend the least amount of energy on temperature regulation in the thermoneutral zone, which is the ambient temperature range where respiration rates do not change with temperature. Within this zone, heat loss is generated with little direct energy expenditure, typically through varying rates of venous blood flow or by altering body posture. Therefore, yawns could provide a metabolically inexpensive means for cooling through countercurrent heat exchange and by increasing cerebral blood flow. Outside of this zone, however, temperature regulation requires increases in metabolism through shivering or panting. For instance when ambient temperature rises above the upper critical temperature, more effective evaporative heat loss mechanisms are increased (Scholander et al., 1950), while other mechanisms are progressively reduced (i.e., yawning). Importantly, follow-up experiments in budgerigars and rats show that yawning is sensitive to both temperature range and direction of change, and not merely elicited by any change (see Gallup et al., 2010b, 2011 for results and detailed discussion).

In the most recent test of this hypothesis, which spurred this review, Gallup and Eldakar (2011) demonstrated that contagious yawning frequency in humans was sensitive to ambient temperatures accompanying seasonal climate variation. Explicitly testing the second prediction of the thermal window hypothesis, i.e., that yawning should decrease when ambient temperatures draw near or exceed body temperature, pedestrians were significantly less likely to yawn in response to viewing images of people yawning at especially hot temperatures (37°C—human body temperature). However, when ambient temperatures were cooler during the winter condition (22°C), pedestrians yawned at the same rate found in laboratory studies at room temperature (Platek et al., 2003). Ambient temperature was the sole predictor of this difference across conditions, while increasing time spent outside further reduced contagious yawning in the summer heat. For instance, individuals who yawned during in the summer reported being outside for less than 5 min, while the average time spent outside of those who did not yawn was over 19 min.

## Evidence and applications of the thermoregulatory theory in medicine and clinical research

### Sleep, yawning, and thermoregulation

It is well documented in humans that yawning occurs most often before sleep onset and after waking (Provine et al., 1987), and similar circadian effects have been demonstrated in laboratory rats (Anias et al., 1984). Sleep and body temperature vary inversely (Gilbert et al., 2004), and in humans yawning frequently occurs in the evening, when brain temperature is at its peak, and upon waking, when brain temperature begins increasing from its lowest point (Landolt et al., 1995). By lowering brain temperature and maintaining thermal homeostasis, the thermoregulatory theory suggests that instead of prompting sleep, yawning actually serves to maintain focus and attention, thereby antagonizing sleep.

Consistent with the thermoregulatory theory, circadian modulation of body temperature alters sleep propensity in humans (Kumar, 2004). Subjective ratings of sleepiness correlate with increases in skin temperature while lying down (Krauchi et al., 1997) and with increases in core body temperature when standing (Krauchi et al., 2005). In addition, hot water consumption increases body temperature as well as sleepiness, while ice intake produces the opposite effect (Krauchi et al., 2006). Furthermore, prolonged sleep deprivation in rats increases deep brain temperature (Everson et al., 1994). Thus, as with yawning, variation in body temperature is associated with corresponding variation in sleepiness and sleep-related fatigue. This research clears up some misconceptions regarding cases of frequent or excessive yawning, and thus has the potential to provide critical insight to sleep medicine.

### Abnormal yawning and thermoregulatory dysfunction

Despite the close association between yawning, sleep, and thermoregulation, excessive yawning is not always indicative of sleep deprivation or sleep disorder and may in fact be an important symptom of an individual's inability to properly maintain thermal homeostasis (Gallup and Gallup, 2008, 2009a,b; Gallup, 2009). Conditions such as multiple sclerosis, migraine headaches, epilepsy, stress and anxiety, and schizophrenia have all been linked to thermoregulatory dysfunction and are often associated with instances of atypical yawning. Excessive yawning is also symptomatic of conditions that increase brain and/or core temperature, such as central nervous system damage, sleep deprivation, and specific serotonin reuptake inhibitors. Conversely, drugs and conditions that produce hypothermia or decreases in temperature suppress yawning (Table 3).

**Table 3:** **How thermoregulatory changes influence yawning in humans: medical conditions, drug use and behavior (modified from Gallup and Gallup, 2008)**.

	**Temperature increase**	**Temperature decrease**	**Yawn enhancement**	**Yawn suppression**
CNS damage^*^	X		X	
Epilepsy^*^	X		X	
Headaches^*^	X		X	
Modafinil^a^^,^^b^	X		X	
Multiple sclerosis^*^	X		X	
Opiate withdrawal^*^	X		X	
Orexin-A^*^	X		X	
SSRIs^*^	X		X	
Sleep deprivation^*^	X		X	
Stress^*^	X		X	
D_2_-like agonists^c^		X		X
Opioid peptides^*^		X		X
Propranolol^d^^,^^e^		X		X
Sleep^*^		X		X

* Reviewed in Gallup and Gallup (2008);

a Launay et al. (2002);

b Gallup and Gallup (2010);

c Collins et al. (2007);

d Meythaler and Stinson (1994);

e *Ghanizadeh (2011)*.

It has therefore been suggested that physicians could use excessive yawning as a diagnostic tool for identifying instances of abnormal thermoregulation (Gallup and Gallup, 2008). Not only is excessive yawning indicative of thermoregulatory dysfunction (Gallup and Gallup, 2010), but yawns can also provide symptom relief in patients suffering from conditions such as multiple sclerosis similar to deliberate behavioral cooling methods (Gallup et al., 2010a). For instance, cooling of the head and neck has been shown to alleviate multiple sclerosis symptoms (Ku et al., 1999) and diminish yawning (Gallup and Gallup, 2007, 2010), supporting the view that yawns provide a temporary brain cooling effect in these patients. Together, this evidence supports the view that frequent yawning associated with various conditions and medications (Table 3) is an adaptive response to counter intermittent brain temperature rises.

### Propranolol in yawning prophylaxis: a case report

Aside from the previously described behavioral cooling methods of nasal breathing and forehead cooling (Gallup and Gallup, 2007, 2010), there has been no evidenced-based treatment for excessive yawning. Recently, however, Ghanizadeh (2011) describes a compelling case study of how the use of propranolol repeatedly extinguished extended periods of frequent yawning in a middle-aged male. Propranolol is a non-selective beta-blocker used to treat hypertension and anxiety, and one side effect is thermoregulatory cooling (e.g., Brittain and Handley, 1967; McSorley and Warren, 1978; Meythaler and Stinson, 1994; Soszynski et al., 1996). For instance, propranolol has been shown to reduce centrally mediated hyperthermia in humans following traumatic brain injury (Meythaler and Stinson, 1994) and prevent increases in body temperature triggered by psychological stress (Soszynski et al., 1996). The above-mentioned evidence supports the view that propranolol reduces yawning frequency through its brain cooling effects (Ghanizadeh, 2011). Research of this nature deserves further attention in clinical studies.

### Yawning and fever

Another testable, and perhaps counterintuitive, implication of the thermoregulatory theory is that yawning should diminish during pyrexia, or fever. As described by Gallup and Gallup (2008, p. 12):

*An elevation of body temperature can occur either due to thermoregulatory failure (hyperthermia), or from intact homeostatic responses such as fever (Simon, 2007). It is well-established that fever is an adaptive host defense mechanism and an essential defensive response to infection by pathogens (Soszynski, 2003). Consistent with this notion* Mariak et al. (1998) *found that brain temperature during fever is not selectively suppressed by any specific thermolytic mechanisms, and research has shown that attempts to treat fever can have harmful effects on critically ill patients, leading to an increase in mortality (Kluger et al., 1996; Schulman et al., 2005). Therefore, the mechanisms that trigger an increase in the thermoregulatory set point (fever) in the hypothalamus may, as a testable implication of our model, override or turn off normal operating thermal mechanisms such as yawning (also in the hypothalamus) to fight the infection*.

## Additional features and predictions of the thermoregulatory theory

### Yawning and boredom

Yawning is often interpreted as an indication of boredom, lack of interest, and sleepiness, and some have even hypothesized that yawning is simply an expression of boredom, unconcern, or indifference (Barbizet, 1958; Baenninger and Greco, 1991). As described by Shoup-Knox (2011), the thermoregulatory hypothesis would alternatively suggest that yawning is a symptom of one of several factors influencing brain temperature, including insufficient blood flow, time of day, and high rates of metabolic heat production. It is therefore predicted that similar to the experience of feeling tired (see above), boredom would also be accompanied by rises in brain temperature.

### Contagious yawning

Due to its contagious nature in a few species of mammals and birds (recently reviewed by Miller et al., 2012b), yawning may also have a more derived social role in some group-living vertebrates. Research on contagious yawning in humans has focused on understanding how individual social characteristics or cognitive development influences its release (e.g., Anderson and Meno, 2003; Platek et al., 2003; Senju et al., 2007; Millen and Anderson, 2010), and a popular view is that contagious yawning is related to empathy. For instance, it was recently observed that humans are more likely to yawn contagiously when they witness kin and friends yawn, in comparison to acquaintances or strangers (Norscia and Palagi, 2011). Since contagion is disrupted by thermal influences (Gallup and Gallup, 2007; Gallup and Eldakar, 2011), however, it is proposed that any derived function of contagious yawning is tied to cognitive processing since hyperthermia impairs cognitive functioning (e.g., Racinais et al., 2008).

Considering that yawning is triggered during low states of vigilance (Guggisberg et al., 2007) and produces changes in localized circulation (Zajonc, 1985; Askenasy, 1989), the spreading of this behavior to nearby conspecifics has been proposed to enhance group vigilance (Gallup and Gallup, 2007). Recent evidence suggests that budgerigars both yawn and stretch contagiously (Miller et al., 2012b) and that the social transmission of these behaviors is enhanced following environmental threats (e.g., auditory disturbances) (Miller et al., 2012a). Consistent with the view that contagious yawning evolved to coordinate arousal, which in turn would improve vigilance within the group, the close behavioral coupling of yawning and stretching among flock-mates may function in the collective detection of, and response to, local disturbances or threats.

## Critiques of the thermoregulatory theory

Despite overwhelming empirical evidence supporting the thermoregulatory theory, some individuals remain skeptical. While theories benefit from criticism and scrutiny that drive more rigorous scientific investigation, unfounded criticisms can hinder progress and delay future applications. Here we address the critiques of the thermoregulation theory of yawning, showing that they remain untenable.

### Yawning cannot cause significant decreases in temperature (Elo, 2010, 2011)

Elo (2010, 2011) has repeatedly argued that yawning cannot cause significant decreases in temperature in the absence of sweating. This conclusion, however, is based solely on calculations referring to changes in the overall body temperature in humans, and not to more localized changes in the specific areas relevant to yawning (i.e., the neck, face, and head). In the case report under scrutiny (Gallup and Gallup, 2008), two patients reported excessive yawning attacks lasting upwards of 45 min in length and immediately following these bouts it was observed that oral body temperatures significantly decreased. During these attacks each yawn consisted of powerful jaw, neck, and body stretching, and time spent in between yawns included deep inhalations. Therefore, the associated respiratory and cardiovascular changes accompanying excessive yawning in these patients could easily contribute to cooling of the skull in the absence of perspiration (see Gallup, 2011a).

Contrary to Elo's position, the thermoregulatory theory has outlined well documented and theoretically founded mechanisms for which the physiological consequences of yawning could produce selective brain cooling (see above). Elo's position is also inconsistent with research directly investigating the relationship between yawning and brain temperature changes in rats (Shoup-Knox et al., 2010; Shoup-Knox, 2011). In particular, the aforementioned reports on rats have shown that isolated yawns produced significant localized brain cooling in the prelimbic cortex and preoptic area of the hypothalamus (Shoup-Knox et al., 2010; Shoup-Knox, 2011). Therefore, the conclusions of Elo (2010, 2011) are invalid.

### The association between yawning and temperature can be explained by other factors (Guggisberg et al., 2010)

In a recent review, Guggisberg et al. (2010) concluded that the association between yawning and temperature was not causal and could be explained by other factors. They attempt to discredit the results of Gallup and Gallup (2007), which showed that methods of behavioral brain cooling (i.e., nasal breathing and forehead cooling) diminish yawning, by arguing that it is impossible to differentiate the effects of temperature and sleepiness (i.e., warmer forehead packs may increase yawning because participants may become sleepy). This argument, however, does not take into account that nasal breathing does not affect sleepiness, and that none of the participants yawned in response to contagious stimuli when inhaling and exhaling through their nose. Therefore, the temperature/sleep confound described by Guggisberg et al. (2010) is untenable.

Guggisberg et al. (2010) also suggest that the association between yawning and rising ambient temperatures reported by Gallup et al. (2009) may be related to rapidly changing vs. stable temperatures, or due to uncontrolled factors such as differences in drowsiness. It has since been shown, however, that yawning is sensitive to both temperature range and direction of change, and not merely elicited by any change (Gallup et al., 2010b, 2011). For instance, yawning in rats is triggered by deviations in thermal homeostasis, and not simply unstable temperatures. In addition, according to Guggisberg et al. (2010) the association between warm temperature and sleep would predict that yawning should be more frequent when ambient temperatures are held high since this would produce an increase in drowsiness or fatigue. However, these expectations run contrary to the thermal window hypothesis, as well as the evidence. Both experimental and descriptive research shows that yawning is significantly reduced at high temperatures, and that this is not an artifact of increased sleep or rest (Gallup et al., 2009, 2011; Gallup and Eldakar, 2011).

### The thermoregulatory theory does not demonstrate any yawn-induced effects (Guggisberg et al., 2011)

According to Guggisberg et al. (2011), contagion is the only effect that is induced by yawning. These authors state that physiological explanations of yawning fail to show a yawn-induced effect, which is argued to support their notion that the “origin and function” of yawning is social (Guggisberg et al., 2010). On the contrary, however, yawn-induced cooling effects have been documented and are outlined throughout this review (Table 1). In addition, Corey et al. (2012) has more recently provided clear evidence of yawn-induced effects on a number of aspects of human physiology.

Furthermore, the results from Gallup and Eldakar (2011), as well as Gallup and Gallup (2007), challenge the social/communication hypothesis as an explanation for the origin of yawning. Contagious yawning was the dependent variable in both of these studies and was significantly, and independently, inhibited by extreme ambient temperatures and methods of behavioral brain cooling. Therefore, counter to the position of Guggisberg et al. (2010, 2011), yawn-induced contagion effects appear to be at least partially mediated by underlying thermoregulatory physiology.

Importantly, neither social contagion nor thermal effects can deny the other. It has been shown that you can change sensitivity to contagion, but it may also be possible to lose a bit of thermoregulatory control under strongly contagious situations. It is critical, however, that any hypothesis on yawning can take into account the existing empirical literature. The social/communication hypothesis fails explain how the “communication” purportedly involved in yawning somehow breaks down at high ambient temperatures or when individuals breathe through their nose.

### The delay in brain cooling following a yawn is too long (Guggisberg et al., 2011)

In discussion of the brain cooling effects in rats following yawning and stretching (Shoup-Knox et al., 2010), Guggisberg et al. (2011) argue that even when considering the time for venous blood drain and thermal convection to occur, the delay observed before brain temperature began to fall is too large to be explained by yawning. In particular, they state, “The thermometer seems to have been placed close to the dura and therefore should have rapidly captured a blood flow induced temperature change” (p. 1303). This argument ignores the fact that yawning occurred during rapid increases in brain temperature (~0.1°C). It is simply unreasonable to suggest that there should not be a slight delay in cooling via increases in blood flow. Not only would there be a delay in reducing a stable temperature, but this would be even greater when counteracting already rising temperatures. Furthermore, the mechanisms of counter-current heat exchange and evaporative effects are not discussed and would require a certain delay before reducing brain temperature. Guggisberg et al. (2011, p. 1303) also state, “… the brain continued to warm up with the same speed as before until ~20–40 s after the yawns and stretches of the observed rats.” This is simply not an accurate depiction. Shoup-Knox et al. (2010) actually showed that prelimbic temperatures began to decrease at an average of 18 s following each isolated yawn, while stretch-induced decreases in prelimbic temperature took 38 s (over twice as long). Furthermore, the rate of increase did in fact slow during this transition. The position of Guggisberg et al. (2011) also fails to account for the discrepancy in cooling effects between yawning and stretching, while the thermoregulatory theory clearly explains this. In short, the argument by Guggisberg et al. (2011) is untenable.

### The thermoregulatory theory overlooks the existence of fetal yawning (Walusinski, in press)

In a review of the theories on the function of yawning, Walusinski (in press) states the thermoregulatory theory has “overlooked” the existence of fetal yawning. Consistent with the view that yawning is phylogenetically old, the onset of this behavior occurs quite early in uterine development (11 weeks gestation in humans) (de Vries et al., 1982). Counter to the views of Walusinski (in press), however, this evidence has indeed been taken into account within the framework of the thermoregulatory model (see Gallup et al., 2009). According to the argument presented by Walusinski (in press), any behaviors that occur in utero should serve identical functions following birth. Thus, because the mother controls thermoregulation of the fetus, Walusinski believes that brain cooling cannot be the function of yawning thereafter. Contrary to this view, however, many important postnatal behaviors begin to appear prenatally (e.g., breathing movements, swallowing and eye movements) before they develop their externally intended functional significance (Nijhuis, 2003). In other words, any functions that prenatal behaviors may have are not necessarily going to match the function(s) to these behaviors following birth. Therefore, the existence of fetal yawning neither supports nor opposes the thermoregulatory theory.

### The thermoregulatory theory overlooks yawning in poikilotherms (Walusinski, in press)

Walusinski (in press) also argues that the thermoregulatory theory could not provide an accurate explaination for yawning in homeotherms because it “overlooks” yawning in **poikilotherms** (i.e., cold-blooded animals). According to this position, he believes there should be a single function to yawning across all vertebrate species. In other words, if poikilotherms such as fish, amphibians, and reptiles yawn, and this is presumably unrelated to maintaining optimal brain temperature, then this behavior should not function in brain thermoregulation among homeotherms either. There are four primary weaknesses in this argument.

First, the discussion of brain cooling in relation to poikilotherms has been mentioned in papers related to the thermoregulatory model (e.g., Gallup et al., 2010b, 2011), and in fact is has been suggested that yawning could play a role in behavioral thermoregulation in these species as well (Gallup et al., 2010b). Yawning is behavioral mechanism of cooling and poikilotherms are particularly dependent on behavioral cooling. Furthermore, many poikilotherms experience the problem of trade-offs in heating, i.e., they benefit from warmer temperatures for digestion but such sustained temperatures may be too high for neural tissue. Exactly what and how yawning cools would depend on the morphology of the brain or areas particularly sensitive to overheating in each species. To our knowledge, no good comparative studies have been performed.

Second, it is important to point out that poikilotherms may not even yawn in the same way that birds and mammals do (see Rasa, 1971; Dullemeijer and Povel, 1972, for explanations of this action pattern in fish and snakes). Baenninger (1987) argues that although amphibians and reptiles open their mouth widely on occasion, this does not necessarily represent yawning.

Third, even if poikilothermic animals do yawn like homeotherms, and yawning were to serve disparate functions from thermoregulation in these species, this does not remove the possibility that yawning has derived thermoregulatory functions in birds and mammals (discussed in Gallup, 2011a,b,c). This type of argument would be similar to positing that since poikilotherms do not yawn contagiously that we should not expect contagion to be present in homeotherms either, yet we certainly do. Evolution is a cumulative process, which has additive effects on traits over time. Thus it is entirely possible that following the evolution of homeothermy, yawning developed brain temperature consequences in birds and mammals (outlined above), which could have then altered selection of this behavior via pressures of thermoregulation in unstable environments.

Fourth, yawning is elicited by numerous stimuli and it has already been suggested that yawning probably serves multiple functions across vertebrates (Gallup, 2011b). As already mentioned, one clear example of this is the distinction between spontaneous and contagious yawning. Therefore, the mere presence or absence of yawning in poikilotherms can neither be used as support for nor against the thermoregulatory theory. Rather, a more thorough investigation of yawning in poikilotherms is needed.

## Conclusions

Unlike many hypotheses on yawning, the thermoregulatory theory has stood up to rigorous testing as well as various critiques. Not only do predicted brain and localized body temperature fluctuations surround yawning events, but yawns are also triggered or inhibited by environmental factors such as ambient temperature manipulation and medical conditions and drugs/medications that directly affect thermoregulation. When the above evidence is taken together (Table 4), the most parsimonious explanation is that yawning is a thermoregulatory mechanism in homeotherms. This evidence has important applications for the current understanding of yawning in medicine and clinical research, and attempts to diminish the significance of this theory could have detrimental effects in delaying treatments and diffusing both basic and practical knowledge. To date, no other theory can explain why contagious yawning in humans would vary with seasonal temperature ranges.

**Table 4:** **Summary of the association between yawning and thermoregulation**.

(1) Brain centers controlling thermoregulation also control yawning
(2) The physiological consequences of yawning facilitate selective brain cooling
(3) Sleep cycles trigger yawning during distinct changes in brain/body temperature
(4) Conditions, drugs, and neurotransmitters affect yawning and temperature in predicted ways^*^
(5) Yawning is inhibited by nasal breathing and forehead cooling^*^
(6) Predicted fluctuations in brain temperature surround yawning events in rodents^*^
(7) Predicted fluctuations in oral temperature surround excessive yawning in humans
(8) Hyperthermic birds yawn sooner following handling-stress^a^
(9) Fluctuations in ambient temperature produce predicted changes in yawn frequency^*^
(10) Yawning is reduced when ambient temperatures near or exceed body temperature^*^

* Indicates that a replication of this specific effect has been demonstrated in an independent sample;

a *Miller et al. (2010)*.

### Conflict of interest statement

The authors declare that the research was conducted in the absence of any commercial or financial relationships that could be construed as a potential conflict of interest.

## Key Concept

Yawning: Behavior characterized by a gaping of the mouth with deep inspiration, followed by a period of apnea, and a shorter expiration.
Thermoregulation: The ability for an organism to keep its brain and body temperature within a very narrow range, even when surrounding temperatures fluctuate.
Homeotherm: An animal species with a relatively high blood temperature that maintains thermal homeostasis primarily through internal metabolic processes (ex: birds and mammals).
Convective heat transfer: Transfer of heat from one place to another by the movement of fluids.
Evaporative cooling: When the vaporization on the surface of a liquid, typically into the surrounding air, cools an object or liquid in contact with it.
Hyperthermia: When the brain/body absorbs or produces more heat than it can dissipate to maintain thermal homeostasis.
Poikilotherm: An animal with a highly variable internal temperature that is determined by the ambient environment (ex: fish, reptiles, amphibians and many invertebrates).
